# Intraarterial contrast-enhanced ultrasound to predict the short-term tumour response of hepatocellular carcinoma to Transarterial chemoembolization with Lipiodol

**DOI:** 10.1186/s12885-021-08867-5

**Published:** 2021-11-02

**Authors:** Jiang Bo, Han Peng, Zhu LianHua, Fei Xiang, Luo YuKun

**Affiliations:** Department of ultrasound, The First Medical Centre, Chinese PLA General Hospital, No. 28 of Fuxing Road, Haidian District, Beijing, 100853 China

**Keywords:** Carcinoma, hepatocellular, Ultrasonography, Chemoembolization, therapeutic, Contrast-enhanced ultrasound

## Abstract

**Background:**

Transarterial chemoembolization (TACE) is an effective locoregional therapy in hepatocellular carcinoma (HCC). However, it is difficult to predict the tumour response (TR) of TACE intraprocedurally. The aim of this study was to predict the TR after TACE (1–3 months) in HCC patients using intraprocedural intraarterial contrast enhanced ultrasound (IA-CEUS).

**Methods:**

In this case-control study, consecutive patients who received TACE in our hospital from September 2018 to May 2019 were enrolled. IA-CEUS was performed before and after TACE. Postoperative contrast-enhanced liver MRI was performed 1–3 months after TACE as the gold standard. According to the modified Response Evaluation Criteria in Solid Tumours (mRECIST), ultrasonic manifestations were compared between the complete remission (CR) group and non-CR group by univariate and multivariate analyses. A logistic predictive model was established and validated, and its diagnostic efficiency was evaluated.

**Results:**

Forty-four patients with sixty-one lesions were enrolled in the study. Multivariate analysis identified, the risk factors as a large lesion diameter (OR: 1.84; 95% confidence interval [CI]: 1.009, 3.080; *P* = 0.020), a larger dimension of non-enhancing area in superior mesenteric artery (SMA)-CEUS than the size in B-mode ultrasound preoperatively (OR: 3.379; 95% CI: 1.346,8.484; *P* = 0.010), presence of corona enhancement in hepatic artery (HA)-CEUS postoperatively (OR: 6.642; 95% CI: 1.214, 36.331; *P* = 0.029), and decreased corona enhancement thickness (per centimetre) postoperatively (OR: 0.025; 95% CI: 0.006,0.718; *P* = 0.025). The area under the receiver operating characteristic curve (AUROC) of the predictive model was 0.904 (95% CI: 0.804, 0.966; *P* < 0.001). The sensitivity, specificity, accuracy, positive predictive value, and negative predictive value were 81.08, 91.67, 85.25, 93.75, and 75.86%, respectively. Leave-one-out cross-validation (LOOCV) showed that the accuracy was 77.05%.

**Conclusions:**

Intraprocedural IA-CEUS can be used to predict the TR in HCC patients after TACE.

## Background

Liver cancer is the sixth most common cancer and the third most common cause of death. Hepatocellular carcinoma (HCC) accounts for 75–85% of liver cancer [1]. Transarterial chemoembolization (TACE) is an effective locoregional therapy in unresectable HCC, especially for Barcelona grade B patients [2], with an objective response rate of 43.6–61.5% [3]. Contrast-enhanced MRI can be performed 1–3 months after TACE to assess the tumour response (TR) based on the modified Response Evaluation Criteria in Solid Tumours (mRECIST) guidelines [4–6].

The significance of the intraprocedural evaluation and prediction of TR lies in their ability to help to determine the end-point of TACE, and thus help determine future treatment and predict prognosis, which is beneficial to patients [7, 8]. However, it is difficult to obtain satisfactory predictive performance regarding TR using digital substraction angiography (DSA) due to its poor resolution of complex fine vessels [9, 10]. As an alternative method, intraoperative cone-beam CT with its higher resolution, has better predictive performance, but a high density of lipiodol deposited within tumours may affect the CT results [11, 12]. In recent years, new imaging methods, such as MRI with new sequences and PET/CT, have been explored to predict TR [13–16], but they are not suitable for intraoperative implementation due to the complexity of the equipment. Therefore, it is imperative to seek an intraoperative method that can be used to accurately predict TR and provide insight regarding TACE endpoints.

As a pure blood-pool imaging method, contrast-enhanced ultrasound (CEUS) is a highly accurate method for displaying tissue microcirculation in real time, providing higher resolution in the microcirculation than DSA, CT and MRI [17–19]. It can be performed via different injection methods, including arterial and peripheral venous routes. Intraarterial contrast-enhanced ultrasound (IA-CEUS) can be performed intraprocedurally via catheters inserted into feeder arteries to observe the arterial supply more selectively and directly than possible via veins, without interference from the surrounding tissues [20]. IA-CEUS has been used to guide TACE and provide information on necrosis [21], but has not been studied with respect to TR prediction. IA-CEUS has the advantage of demonstrasting arterial blood supply in tumours; intra-hepatic arterial CEUS can only observe the blood supply of the arterial phase of the tumour, while intra-superior mesenteric artery CEUS can only observe that of the portal vein, and the interference between the arterial phase and the portal vein phase is less than that of intravenous CEUS. We speculated that IA-CEUS has good performance in evaluating and predicting TR. The aim of this study was to explore the clinical value of IA-CEUS in predicting TR after TACE at 1–3 months.

## Methods

This single-centre study was approved by the Ethics Committee of the Chinese People’s Liberation Army General Hospital (Approval No. S2020–427-01). All the patients provided written informed consent.

### Study population

Consecutive patients who received TACE treatment at PLA General Hospital from September 2018 to May 2019 were enrolled in this study.

**Fig. 1:**
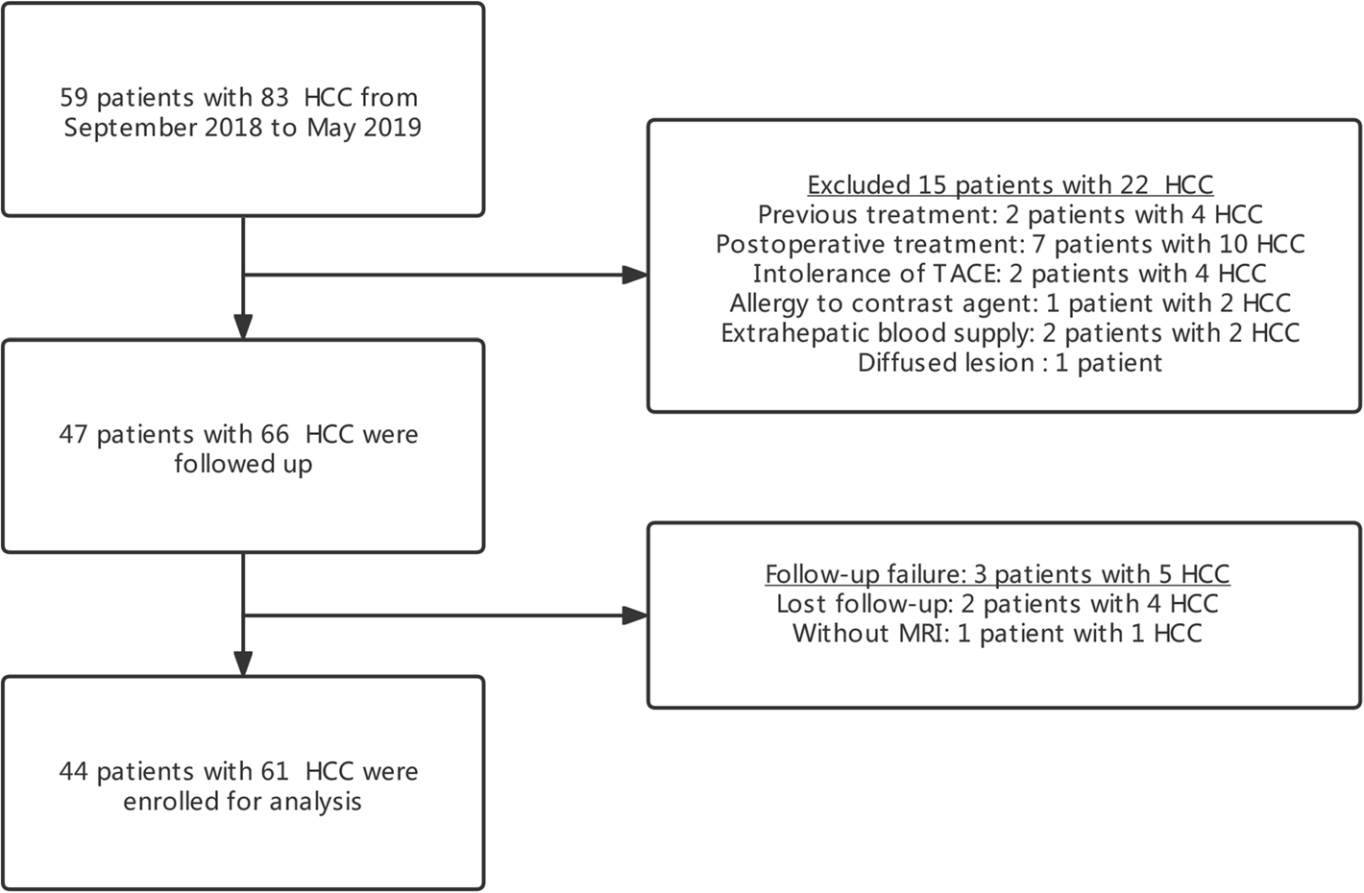
Flowchart of patient enrollment. TACE = transarterial chemoembolization, HCC = hepatocellular carcinoma

The inclusion criteria were as follows: (a) the patient met the diagnostic criteria of the European Association for the Study of the Liver [2]; (b) the patient conformed to the TACE treatment indications and successfully completed the TACE operation; (c) the tumour that had not been treated previously; (d) the tumour that could be detected, located and recorded by ultrasound (US) or CEUS in a horizontal position; and (e) the patient had a life expectancy of longer than six months. The exclusion criteria were as follows: (a) the lesion received treatment during follow-up; (b) the patient had uncontrolled hypertension (> 140/90 mm/Hg); heart, respiratory, liver or renal failure or severe bleeding diathesis that could not tolerate TACE; (c) the patient had allergies to US contrast agent; (d) the hepatic artery (HA) and superior mesenteric artery (SMA) originated from the same trunk or extrahepatic arteries supplied the tumour; (e) the lesions were diffuse (> 70% of liver volume); (f) the patient had portal thrombus or distant metastasis; and (g) the patients failed to be followed up with contrast-enhanced MRI or the results were not available. The details of patient selection are listed in Fig. 1.

The clinical characteristics of the patients, including sex, age and hepatitis background, were recorded before TACE.

### Conventional TACE procedure

All TACE procedures were performed by two experienced interventional radiologists (with 15 and 5 years of TACE experience) in a consistent way. After local anaesthesia, the right femoral artery was punctured with the modified Seldinger technique. A 4-Fr arterial catheter (RH, Terumo, Japan) was inserted for selective HA and SMA, and then a 3-Fr microcatheter (Progreat, Terumo, Japan) was superselectively inserted into the supply artery of the tumour. An oil-in-water-based emulsion comprising chemotherapy drugs epirubicin (30–50 mg), oxaliplatin (100–150 mg), 5-fluorouracil (5-FU) (500–750 mg) and leucovorin (200–300 mg) mixed with lipiodol (8 ml) at a proportion of 1:1 were injected through a microcatheter, and then 0.1 g polyvinyl alcohol (PVA) particles (300–500 μm) and gelatine sponge particles were injected successively. The treatment endpoint was determined by the interventional radiologist, and the main manifestation was the complete coverage of the tumour site by lipiodol deposition and feeding artery shown as a “dry-branch”, which means there were no blood supply absolutely.

### US technique

All US examinations were performed by one experienced US physician (FX, with more than 10 years of experience in liver CEUS) using a Siemens Oxana2 or S2000 ultrasonic system equipped with CEUS software. The transducer was a C6–1 convex array transducer operated at 3.5–5.0Mz. All of the parameters were adjusted to ensure high-quality images. Before TACE, B-mode US was performed to define quantity (single or multiple), diameter (measured on the maximum section), location (lobe), and echogenicity (hypoechoic, isoechoic or hyperechoic) of the lesions. The gain, depth, focus, and dynamic range were appropriately adjusted. Although each patient had multiple nodules, specific nodules were selected for observation. The inclusion criteria for the nodules were (a) a diameter less than 10 cm; (b) a depth of no more than 8 cm from the skin; and (c) a good acoustic window for clearly display by US.

### IA-CEUS technique

IA-CEUS, including hepatic artery contrast-enhanced ultrasound (HA-CEUS) and superior mesenteric artery contrast-enhanced ultrasound (SMA-CEUS), was performed using an arterial catheter inserted before TACE, while HA-CEUS was performed again immediately after TACE. The time interval between IA-CEUS and TACE was less than 10 min. During HA-CEUS, the contrast agent reached the tumour directly through the supplying artery. There was no contrast agent in the portal vein, so the tumour only had enhancement of the arterial phase. In SMA-CEUS, the contrast agent reached the tumour through the portal vein, so the portal vein supply of the tumour was shown alone without disturbance of the arterial phase.

Contrast pulse sequence mode was selected for image acquisition. Before performing CEUS, the lesions were reconfirmed in B-mode US. The mechanical index was set in the range of 0.06 to 0.1. The focus was set at the bottom of the screen. The gain and depth were adjusted accordingly. The contrast agent was sulphur hexafluoride (Sonovue®, Bracco, Milan Italy). The contrast agent for SMA-CEUS was dissolved according to the specification (mixed with 5 ml normal saline to form a suspension), and the bolus injection dose was 1.5 ml. The concentration of contrast agent in HA-CEUS was 0.02 ml/ml (the SMA-CEUS contrast agent was diluted using normal saline to a concentration of 1:50), and the injection dose was 4 ml (at a speed of 1 ml/second). Continuous storage of the dynamic images spanned no less than 3 min.

### Imaging evaluation

After TACE and IA-CEUS, IA-CEUS images were retrospectively evaluated frame-by-frame offline by two experienced US physicians (J.B. and H.P., with more than 5 years of experience, did not participate in TACE). An image was excluded if it was considered to be of poor quality by both doctors. When there was a disagreement between them, they discussed it together until a consensus was reached.

**Fig. 2:**
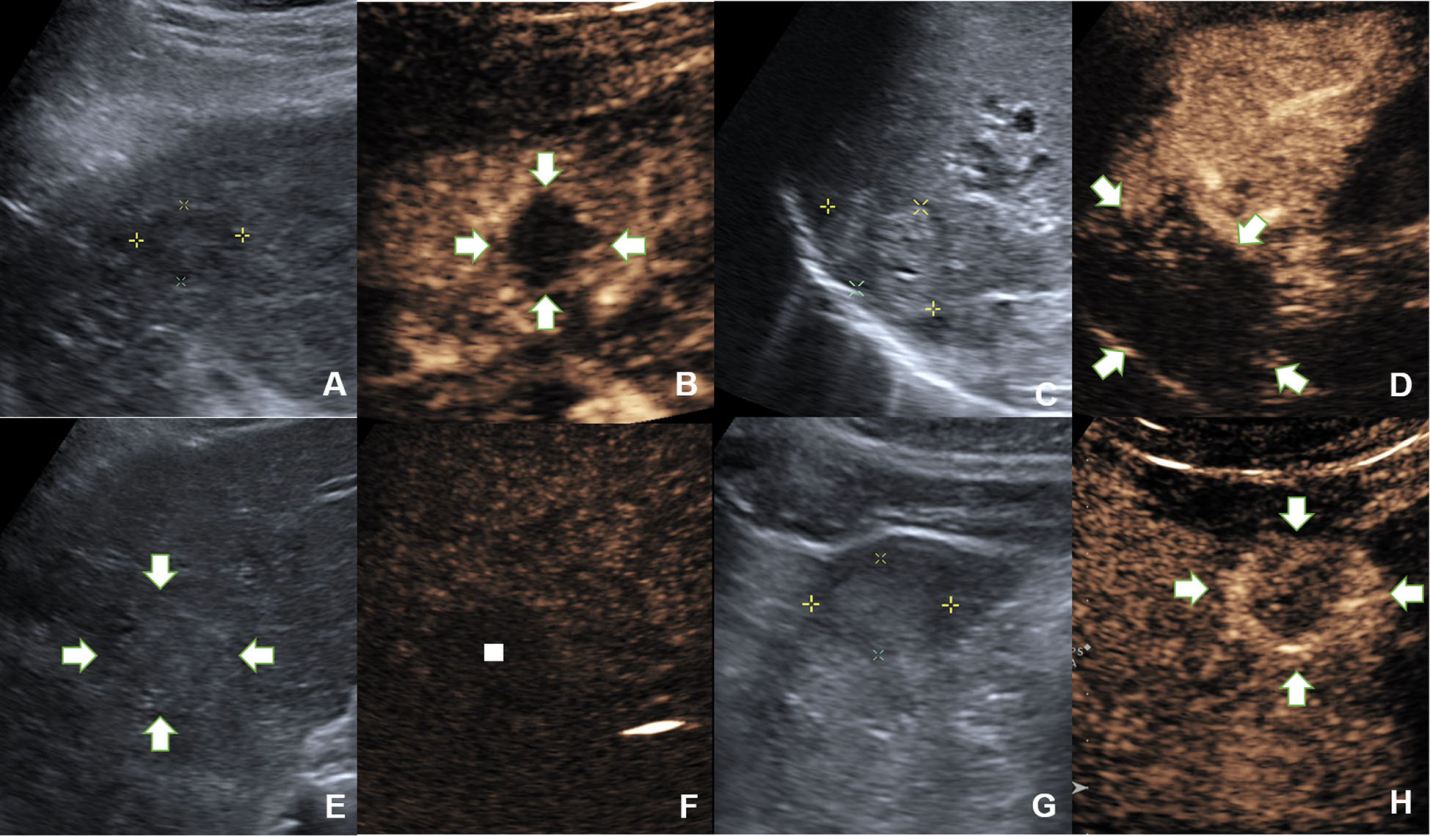
Examples of image evaluation in IA-CEUS. **A** & **B**, A hypoechoic lesion without intratumoural enhancement in SMA-CEUS preoperatively, the dimension of non-enhancing area (indicated by the arrow) was equal to the size in B-mode ultrasound. **C** & **D**, A hyperechoic lesion without intratumoural enhancement in SMA-CEUS preoperatively, the dimension of non-enhancing area (indicated by the arrow) was larger than the size in B-mode ultrasound. **E** & **F**, In preoperative HA-CEUS, there was no corona enhancement around the tumour (indicated by the arrow in B-mode and by rectangle in HA-CEUS) in late enhancing phase. **G** & **H**, In preoperative HA-CEUS, corona enhancement can be seen around the tumour (indicated by the arrow) in late enhancing phase. HA-CEUS = hepatic artery contrast-enhanced ultrasound; SMA-CEUS = superior mesenteric artery contrast-enhanced ultrasound

Parameters observed in HA-CEUS before and after TACE included intratumoural enhancement (presence or absence), corona enhancement in the late phase (presence or absence) and corona thickness (measured at the thickest point). The decrease in corona thickness after TACE was calculated. The parameters observed in SMA-CEUS before TACE included intratumoural enhancement (presence or absence) and the dimension of non-enhancing area (compared with B-mode ultrasound in the same section). The result of dimension of non-enhancing area can be divided into larger non-enhancing area and no larger non-enhancing area. Examples can be seen in Fig. 2.

### Follow-up

Contrast-enhanced liver MRI was performed as the gold standard of TR for all patients to evaluate whether the nodules had a blood supply 1–3 months after TACE. Images were analysed by two abdomen MRI radiologists (with more than 10 years of experience) who were blinded to the IA-CEUS results. The result of TR in this study was evaluated on the bases of nodule rather than patient. Lesions were divided into a complete remission (CR) group and a non-CR group according to the modified response evaluation criteria in solid tumours (mRECIST) standard [2, 5]. CR was defined as the detection of the target nodule without any intratumoral arterial enhancement. Non-CR was defined as the detection of the target nodule with residual intratumoral arterial enhancement. The diagnoses of the two radiologists were compared, and when the results were inconsistent, a consensus was achieved after discussion and was regarded as the diagnosis.

### Statistical analysis

All data were separately recorded by two researchers (Z.L.H. and H.P.), and then the accuracy was evaluated. For data following a normal distribution, continuous variables were expressed as mean values and standard deviations, and the independent t-test was used to analyse differences. For data following a nonnormal distribution, continuous variables were expressed as medians and interquartile ranges. and the Mann-Whitney U-test was used to analyse differences. Categorical variables were represented as frequencies and percentages, and independent or modified χ^2^ tests were used to analyse differences. After univariate analyses, variables with statistical significance were subjected to logistic regression. *P* < 0.05 was considered statistically significant. The logistic predictive model was established based on the multivariate analysis and the Hosmer-Lemeshow goodness-of-fit test and leave-one-out cross-validation (LOOCV) were performed to validate the model. The receiver operating characteristic (ROC) curve was obtained, and the sensitivity, specificity, accuracy and area under the ROC curve (AUROC) were calculated. The data were analysed by using the statistical software SPSS 21.0 (IBM, Somers, NY).

## Results

### Characteristics of the patients & tumours

As presented in Table 1, forty-four patients were enrolled in the study, comprising 41 males (93.18%) and 3 females (6.82%). The average age of the patients was 58.64 ± 11.26 years, and the range was 32 to 79 years. All the patients had multiple nodules. After standard selection, sixty-one lesions were enrolled for observation. The mean lesion diameter was 3.79 ± 2.74 cm, with lesion diameter ranging from 0.7 cm to 12.5 cm. Median follow-up time was 37 days, interquartile interval was 33 days to 45 days. Contrast-enhanced MRI results showed that 24 tumours (39.34%) responded and achieved CR, while 37 tumours (60.66%) were recorded as non-CR (comprising 21 tumours with partial remission, 10 with stable disease and 6 with progressive disease). The kappa value of the two radiologists interpreting the MRI results was 0.908. There were no adverse events associated with IA-CEUS during the study.

**Table 1:** Patient and nodule characteristics

characteristics	Number
Patients	
number	44
age(y)^a^	58.6 (11.26)
Sex	
male	41 (93.18)
female	3 (6.82)
Hepatitis background	
hepatitis B	38 (86.36)
hepatitis C	3 (6.82)
hepatitis B + C	1 (2.27)
no hepatitis	2 (4.55)
Lesions	
number	61
maximum diameter (cm) ^a^	3.79 (2.74)
Location	
left lobe	24 (39.34)
right lobe	37 (60.66)
Echo in B-mode ultrasound	
hypoechoic	24 (39.34)
isoechoic	14 (22.95)
hyperechoic	23 (37.70)
MRI result	
complete remission	24 (39.34)
non-complete remission	37 (60.66)
kappa value of two doctors	0.908

Note. — Data in parentheses are percentages unless otherwise indicated

a: Data in parentheses are standard deviations

### Inter-observer agreement of the IA-CEUS characteristics

For HA-CEUS and SMA-CEUS, the observers obtained identical results in terms of intratumoural enhancement, and judgment of whether the dimension of non-enhancing area in SMA-CEUS was larger than the size in B-mode ultrasound. The kappa value was 0.902 for the evaluation of corona enhancement for HA-CEUS before and after TACE.

### Univariate analyses for short-term TR

For HA-CEUS, all the lesions showed hyperenhancement before TACE, while only four lesions showed the presence of intratumoural enhancement in SMA-CEUS. There were no statistically significant differences between the two groups in these two parameters (*P* = 1.00).

As shown in Table 2, the difference in tumour diameter between the CR group and the non-CR group was significant (*P* < 0.001). There was no significant difference in age (*P* = 0.18), sex (*P* = 0.61), hepatitis background (*P* = 0.41), nodule location (*P* = 0.94) or echo(*P* = 0.85) between the two groups.

**Table 2:** Result of univariate analyses for short-term postoperative tumour response

Characteristics	Complete remission (*n* = 16)	non-complete remission (*n* = 28)	*P value*
Patients			
Age(y)^a^	61.69 (9.58)	56.89 (11.92)	0.18
Sex			
Male	14 (31.82)	27 (61.36)	0.61
Female	2 (4.55)	1 (2.27)	
Hepatitis background			
Hepatitis B	14 (31.82)	24 (54.55)	0.41
Hepatitis’s C	1 (2.27)	2 (4.55)	
Hepatitis B + C	1 (2.27)	0 (0)	
No hepatitis	0 (0)	2 (4.55)	
Lesions	CR (*n* = 24)	Non-CR (*n* = 37)	
Diameter (cm) ^a^	2.50 (1.08)	4.63 (3.16)	<0.001
Location			
Left lobe	5 (8.20)	8 (13.11)	0.94
Right lobe	19 (31.15)	29 (47.54)	
Echo in B-mode ultrasound			
Hypoechoic	10 (16.39)	14 (22.95)	0.85
Isoechoic	6 (9.84)	8 (13.11)	
Hyperechoic	8 (13.11)	15 (24.59)	
Preoperative IA-CEUS			
HA-CEUS			
Corona enhancement in late phase			
Yes	22 (36.07)	27 (44.26)	0.14
No	2 (3.28)	10 (16.39)	
Corona thickness (cm) ^a^	0.56 (0.23)	0.67 (0.61)	0.31
Non-enhancing area in SMA-CEUS			
larger than 2D-US	8 (13.11)	22 (36.07)	0.046
No larger than 2D-US	16 (26.23)	15 (24.59)	
Postoperative HA-CEUS			
Intratumoural enhancement			
Yes	12 (19.67)	31 (50.82)	0.005
No	12 (19.67)	6 (9.84)	
Corona enhancement in late phase			
Yes	4 (6.56)	21 (34.43)	0.004
No	20 (32.79)	16 (26.23)	
Corona thickness (cm)^b^	0.00 (0.00, 0.00)	0.30 (0.00, 0.70)	0.003
Corona thickness decrease (cm) ^b^	0.50 (0.40, 0.70)	0.20 (0.00, 0.40)	0.006

Note. — Data in parentheses are percentages unless otherwise indicated. *CEUS* contrast-enhanced ultrasound, *IA-CEUS* intra-arterial contrast-enhanced ultrasound, *HA-CEUS* hepatic artery contrast-enhanced ultrasound, *SMA-CEUS* superior mesenteric artery contrast-enhanced ultrasound. a: Data in parentheses are standard deviations. b: Data are median, with interquartile range in parentheses

**Fig. 3:**
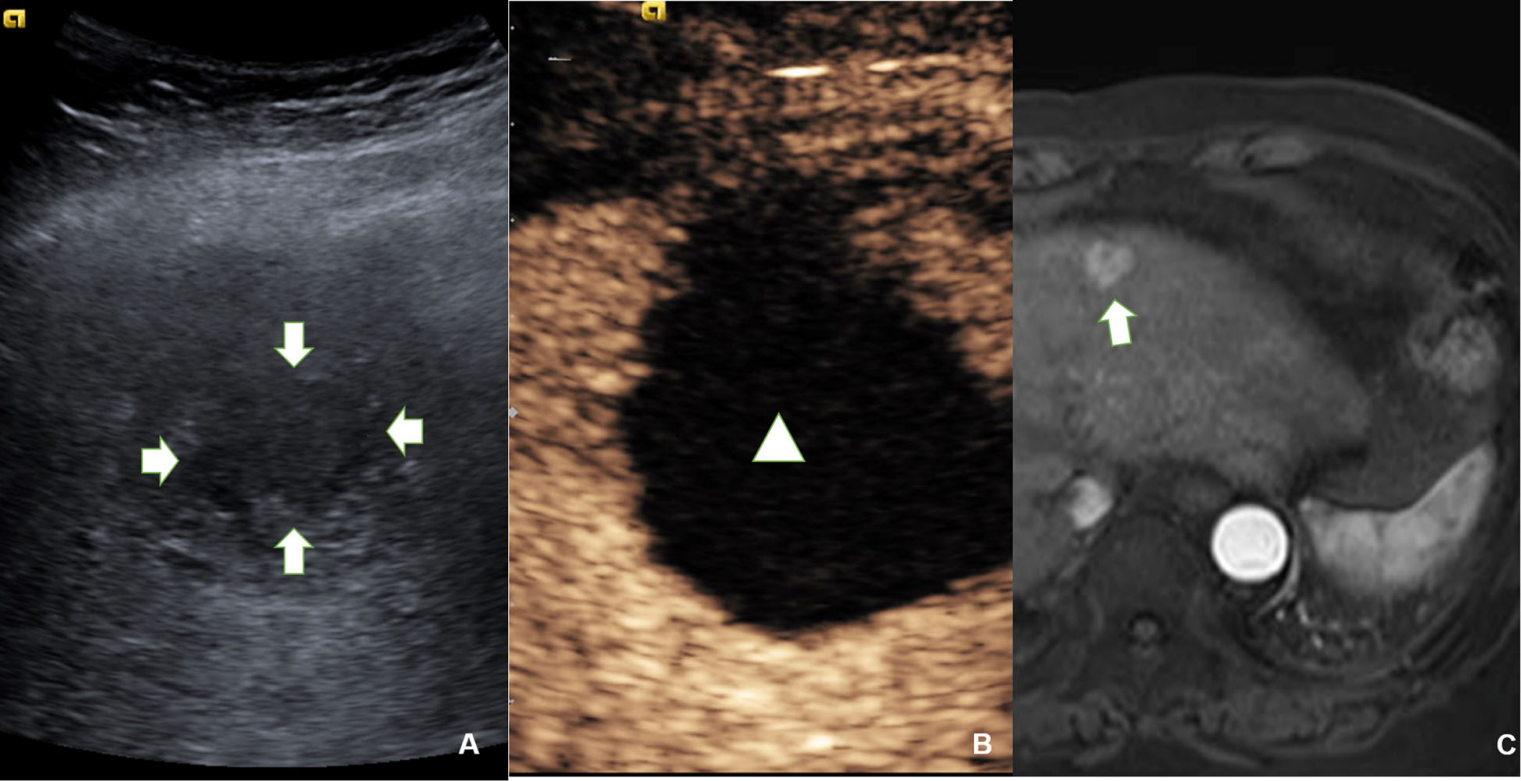
Relationship between a larger dimension of non-enhancing area in preoperative SMA-CEUS and tumour response after TACE. **A**, B-mode ultrasound showed a hypoechoic HCC lesion located in segment 4, which was 3.3 × 3.5 cm (indicated by the arrow). **B**, Preoperative SMA-CEUS showed that a significant larger dimension (3.9 × 4.6 cm) of non-enhancing area (triangle shown) than the size in B-mode ultrasound. **C**, Postoperative abdominal contrast-enhanced MRI showed that the tumour response should be defined as stable disease (indicated by the arrow). TACE = transarterial chemoembolization, HCC = hepatocellular carcinoma; CEUS = contrast-enhanced ultrasound; SMA-CEUS = superior mesenteric artery contrast-enhanced ultrasound

Among the parameters of preoperative IA-CEUS, in the incomplete remission group, the non-enhancing area of SMA-CEUS was more likely to be larger than the size of the tumour shown in B-mode US (larger dimension of non-enhancing area in SMA-CEUS, shown in Fig. 3), the difference was significant (*P* = 0.046). There were no significant differences in corona enhancement (*P* = 0.14) or corona thickness (*P* = 0.31) between the two groups before TACE.

**Fig. 4:**
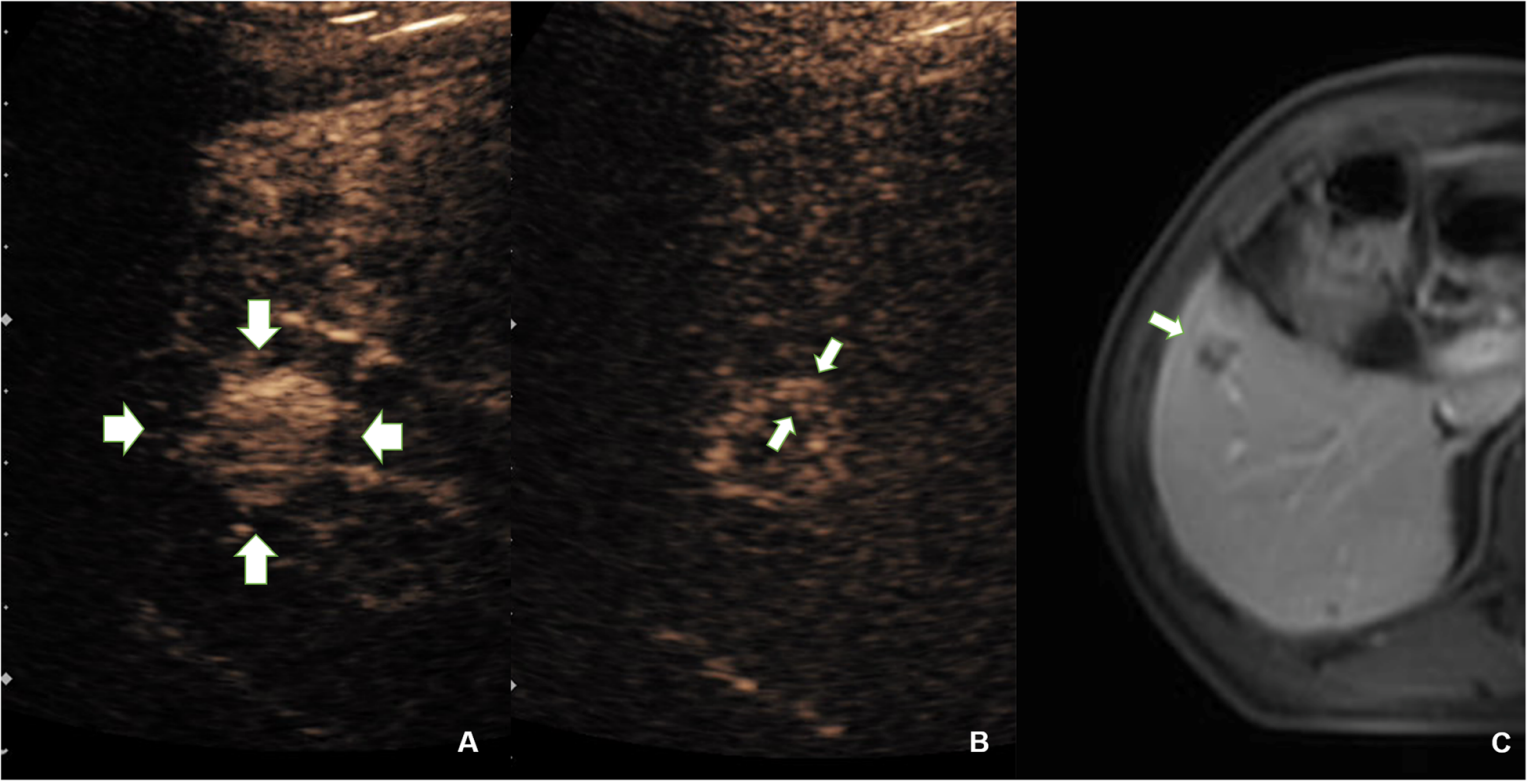
Relationship between presence of corona enhancement in late phase in HA-CEUS and tumour response after TACE. **A**, Preoperative HA-CEUS revealed a hyperenhanced HCC lesion located in segment 8 (indicated by the arrow). **B**, Postoperative HA-CEUS showed corona enhancement in the late enhancing phase. **C**, Postoperative abdominal contrast-enhanced MRI showed a small amount of intratumoural enhancement and peritumoural corona enhancement in portal phase, and the tumour response was defined as partial remission (indicated by the arrow). TACE = transarterial chemoembolization, HCC = hepatocellular carcinoma; CEUS = contrast-enhanced ultrasound; HA-CEUS = hepatic artery contrast-enhanced ultrasound

**Fig. 5:**
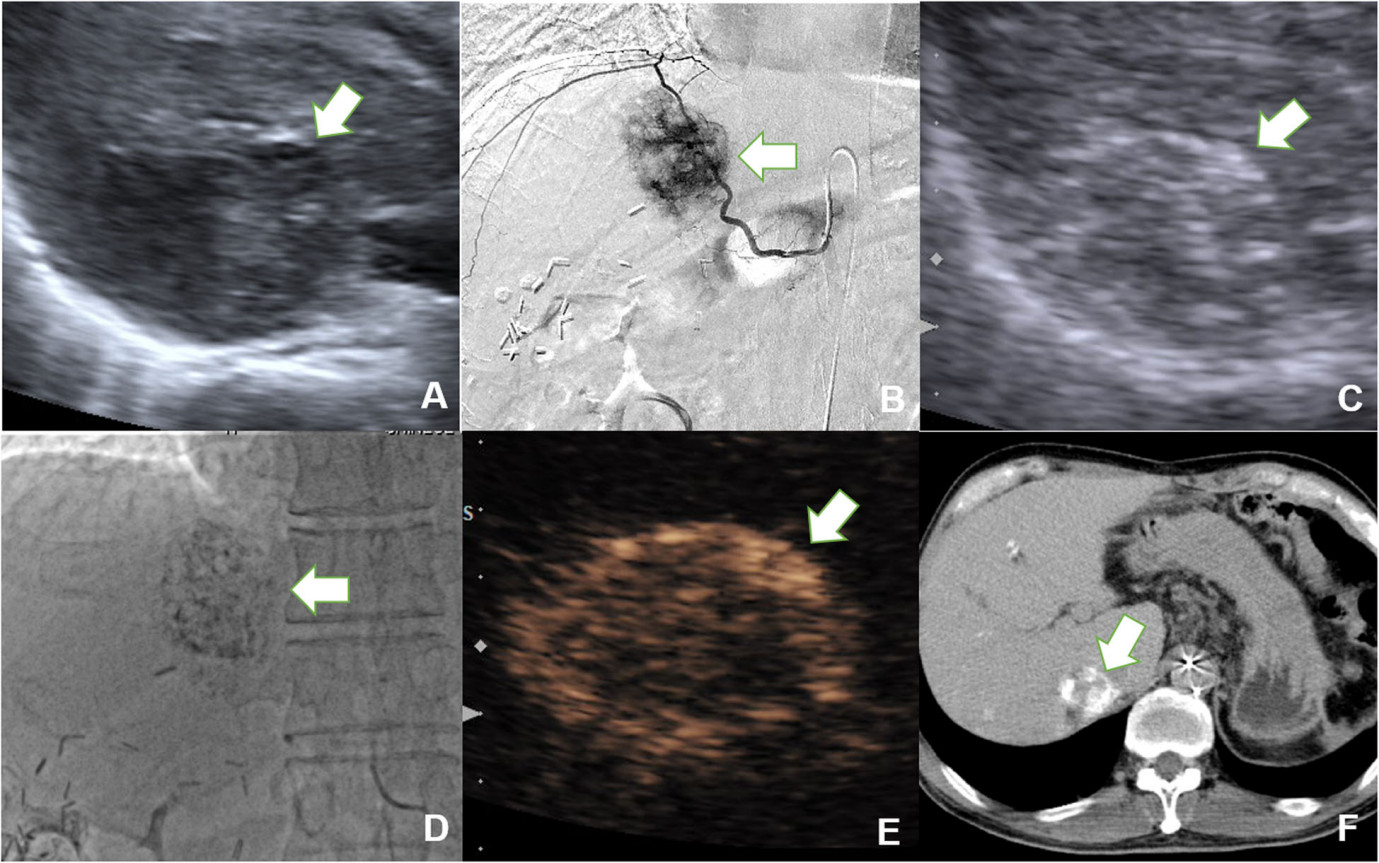
Comparison among HA-CUES, CBCT and DSA imaging. **A**, B-mode ultrasonography showed a hypoechoic HCC lesion located in segment 7 before TACE (arrow). **B**, Supperselected DSA showed the lesion before TACE (arrow). **C**, B-mode ultrasonography revealed lipiodol deposition in the lesion immediately after TACE (arrow). **D**, The lesion was shown on DSA after TACE (arrow). **E**, Postoperative HA-CEUS showed corona enhancement in the late phase (arrow). **F**, Postoperative cone-beam CT showed the high density deposition inside and around the tumour (arrow). HA-CEUS = hepatic artery contrast-enhanced ultrasound; DSA = digital subtraction angiography

After TACE, intratumoural enhancement (*P* = 0.005), corona enhancement (*P* = 0.004) (shown in Figs. 4 and 5), corona thickness(*P* = 0.003), and the decrease in corona enhancement thickness (*P* = 0.006) showed significant differences between the two groups.

### Logistic regression and its goodness-of-fit and accuracy

Due to the clinical association between the presence of corona enhancement and corona thickness after TACE, the latter was excluded from the logistic regression. Table 3 presents the results of the multivariate analyses. Tumour showing a 1 cm increase in diameter were associated with a 1.840-fold (95% confidence interval [CI]: 1.009, 3.080; *P* = 0.020) risk of a poor response. Preoperatively, tumours with an larger non-enhancing area in SMA-CEUS had a 3.379-fold (95% CI: 1.346, 8.484; *P* = 0.010) risk of a poor response. Postoperatively, the presence of corona enhancement in the late phase was associated with a 6.642-fold (95% CI: 1.214, 36.331; *P* = 0.029) risk of a poor response, and this risk was reduced by 6.8% (95% CI: 0.006, 0.718; *P* = 0.025) for every 1 cm reduction in the corona enhancement.

**Table 3:** Result of multivariate logistic regression analyses for short-term postoperative tumour response

Characteristics	B	S. E	Walds χ^2^	*P* value	Odds ratio
Diameter	0.610	0.263	5.373	0.020	1.840 (1.009,3.080)
larger non-enhancing area in SMA-CEUS	1.217	0.470	6.717	0.010	3.379 (1.346,8484)
Postoperative intratumoral enhancement	1.185	0.844	1.972	0.160	3.271 (0.626,17.101)
Postoperative corona enhancement	1.893	0.867	4.769	0.029	6.642 (1.214,36.331)
Corona thickness decrease	−2.692	1.205	4.995	0.025	0.068 (0.006,0.718)
Constant	−4.218	1.476	8.164	0.004	0.015

Note. — Data in parentheses are 95% CIs. *SMA-CEUS* superior mesenteric artery contrast-enhanced ultrasound

**Fig. 6:**
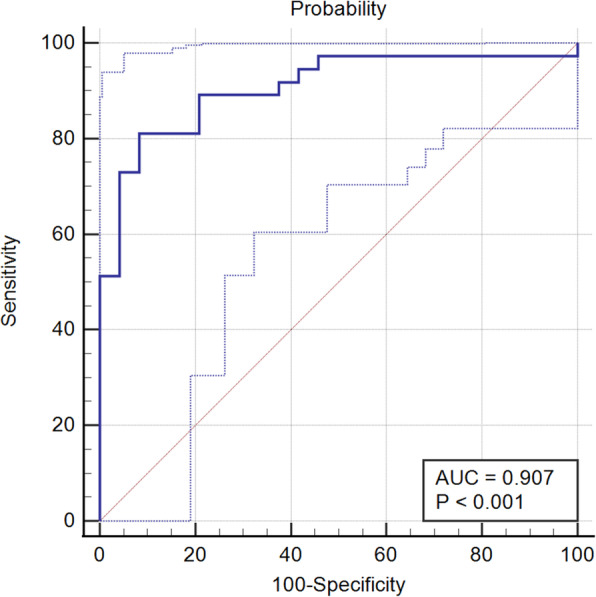
The receiver operating characteristic curve of the predictive model. The area under the curve (solid line) and its 95% confidence interval (dotted line) are shown

A logistic predictive model was established, and the AUROC was 0.909 (95% CI: 0.807, 0.967; *p* < 0.001). The ROC curve is shown in Fig. 6. When the cut-off value was set to the predicted probability of 0.73, the diagnostic sensitivity was 81.08% (30/37), (95% CI: 64.29, 91.44%), the specificity was 91.67% (22/24), (95% CI: 71.53, 98.54%), the accuracy was 85.25% (52/61), the positive predictive value was 93.75% (30/32), (95% CI: 77.78, 98.91%), the negative predictive value was 75.86%(22/29), (95% CI: 56.08, 88.98%), the positive likelihood ratio was 4.29(30/7), and the negative likelihood ratio was 0.091(2/22). The Hosmer-Lemeshow goodness-of-fit test showed that at 8 degrees of freedom, the predicted TR probability could be compared with the actual TR by χ2 test statistics. The results (X2 = 3.30, *P* = 0.91) indicated that the model fit the data adequately. LOOCV was performed to verify the diagnostic efficacy of the model, and the results showed that the model accuracy was 77.05% (47/61).

## Discussion

Early prediction of the response of hepatocellular carcinoma to transarterial chemoembolization is very important; however, intraoperative methods that can be used for accurate evaluation are rare. Our study shows that intraarterial contrast-enhanced ultrasound could sensitively detect intratumoural microcirculation changes and can identify risk factors for poor short-term tumour response after TACE. The established model may assist in treatment response prediction.

The reasons for choosing IA-CEUS are as follows. Firstly, TACE is a local treatment of tumours via blood vessels and the therapeutic effect is achieved by changing the tumour microcirculation. IA-CEUS can display the blood supply of the tumour more directly via arterial catheter. Moreover, HA-CEUS can eliminate the interference of peripheral tissue enhancement and display the wash-out direction of the tumour better to evaluate the distribution of the wash-out blood around the tumour, which may predict the possibility of recurrence or metastasis around the tumour [20]. In addition, since arteries can be selected, HA-CEUS shows only the arterial blood supply, while SMA-CEUS shows only the portal vein blood supply, which may avoid interference between the arterial and portal phases in peripheral vein CEUS [22, 23]. Therefore, the changes in microcirculation after TACE may be shown more specifically by IA-CEUS than conventional CEUS.

Our results showed that larger dimension of non-enhancing area in SMA-CEUS preoperatively may predict poor TR. As shown in Fig. 3, the dimension of portal blood supply loss is larger than the size shown in the B-mode ultrasound. This pattern can be explained by the changes in the HCC microcirculation. Changes in the microcirculation with HCC progression have been demonstrated by Kitao [24]. With the progression of HCC, abnormal arterial hyperplasia leads to an increase in tumour pressure, which causes the hepatic venous system to collapse, initially due to compression. The increasing pressure gradient of blood pressure leads to the gradual conversion of the portal vein from a supplying artery to a draining vessel. An artery-portal shunt develops gradually [25]. The larger dimension of non-enhancing area in SMA-CEUS may indicate that the tumour’s portal system plays a role of drainage rather than perfusion. This type of tumour may have a higher degree of progression than those without larger dimension of non-enhancing area or those still have portal vein perfusion. Moreover, the formation of an artery-portal shunt may make it easier for chemotherapeutic agents to be gradually drained after treatment, rather than remaining inside the tumour for a long time, leading to poor TR. Duran et al. reported that the intratumoural drug concentration 3 weeks after treatment is an influential factor for antitumour effects [26]. Therefore, the combination of artery-portal shunts and portal drainage may decrease the concentration of drugs, leading to poor antitumour effects. However, thus far, we have not found any reports of the use of SMA-CEUS to evaluate the TR of TACE. We believe that this technology may serve as a new tool for evaluating the short-term efficacy of TACE for HCC.

We found that the presence of a corona enhancement postoperatively may lead to poor TR. As is shown in Figs. 4 and 5, the tumoral corona enhancement is shown in the late phase, and the peripheral area of the tumour remained active for 1–3 months after TACE. These corona enhancements may be related to the drainage area of the tumour. According to the theory of Kitao [24], blood flow from the tumour drains into the surrounding hepatic sinuses and forms the drainage area, which is characterized by continuous corona enhancement around the tumour in the late-enhancing phase [27]. The presence of a drainage area indicates that the perfusion of blood flow is still occurring inside the tumour. The accumulation of lipiodol in the tumour may interfere with the operator’s judgement of intratumoural enhancement. Similar conclusions have been reported in previous studies [11]. Intratumoural enhancement may not be accurate enough to adequately evaluate treatment outcomes. In this study, the drainage area was characterized by corona enhancement around the tumour, which was not affected by hyperechoic lipiodol. Moreover, the drainage area was located downstream of the entire microcirculation and played a role in convergence. Therefore, the presence of a drainage area suggested that the HCC was not completely embolized, although there may be no specific enhancement or it may be indeterminate due to the interference of lipiodol. As shown in Fig. 5, the drainage area shown in HA-CEUS was not shown in DSA even though the tumour had met the TACE endpoint in DSA. Even if this phenomenon is demonstrated by CBCT in this case, the consistency of CBCT and CEUS in the display of drainage areas needs further study. In our previous study, we found that the consistency between the drainage area and short-term TR, as evaluated by MRI, was good [28]. In addition, a decrease in drainage area can be explained by a decrease in blood supply within the tumour. Previous studies have demonstrated that a reduction in intratumoural perfusion can predict the efficacy of TACE, consistent with our results [29]. In contrast, some studies have shown that a 35 to 85% reduction in blood supply is better for patient survival [8]. Although we did not conduct any quantitative measurements, the results of the present study tend to support a complete embolization of blood flow.

The results also showed that lesion diameter was an independent risk factor for poor TR after TACE. Watanabe et al. found that tumour size was a risk factor for poor prognosis, consistent with our results [30–32], and diameter reflects tumour progression. Tumours with larger diameters are more progressive and invasive than those with smaller diameters. In addition, larger tumours have abundant arteries and more chaotic vascular distribution [10], which are difficult to block completely.

Our predictive model suggests several risk factors for poor TR of HCC nodules after TACE. The AUROC of the established model reached 0.909 and achieved an accuracy of approximately 77% was achieved in cross-validation, with the model showing similar diagnostic efficacy to those of other studies [33]. Moreover, IA-CEUS can be performed conveniently and quickly during TACE, and there is no need to consider the side effects of repeated injection of contrast agent on liver and kidney function. The obtained results can be used as a reference for interventional radiologists in deciding the endpoint of TACE, which has potential clinical application value.

Our research has two main limitations. First, some of the results have not been confirmed by pathology, especially for the larger non-enhancing area in SMA-CEUS, and corresponding histological studies should be carried out. Second, this study lacks external validation and has a small sample size.

In conclusion, IA-CEUS is an accurate intraprocedural method that can depict the microcirculation of HCC. This technique reflects the characteristics of HCC perfusion and drainage, which are of great significance for the prediction of short-term efficacy after TACE. This established model based on the parameters of IA-CEUS can predict the short-term TR of TACE patients.

## Declarations

Based on Contrast-Enhanced Ultrasound Liver Imaging Reporting and Data System (LI-RADS) guidelines, the clinical application of CEUS was expanded in this study. Although most of the contrast agents were used intravenously in previous studies, contrast agents were used in this study for imaging in the blood pool and did not cause adverse reactions. A similar method was used in a previous study to observe the microcirculation of tumours [20].

## Data Availability

The datasets used and analysed during the current study are available from the corresponding author on reasonable request.

## Abbreviations

HCC: Hepatocellular carcinoma
TACE: Transarterial chemoembolization
TR: Tumour response
DSA: Digital subtraction angiography
CEUS: Contrast-enhanced ultrasound
IA-CEUS: Intra-arterial contrast-enhanced ultrasound
HA: Hepatic artery
SMA: Superior mesenteric artery
